# Materials for Hip Prostheses: A Review of Wear and Loading Considerations

**DOI:** 10.3390/ma12030495

**Published:** 2019-02-05

**Authors:** Massimiliano Merola, Saverio Affatato

**Affiliations:** Laboratorio di Tecnologia Medica, IRCCS—Istituto Ortopedico Rizzoli, Via di Barbiano, 1/10 40136 Bologna, Italy; merola@tecno.ior.it

**Keywords:** biomaterials, ceramic, friction, hip, implants, polyethylene, prosthesis, simulator, wear

## Abstract

Replacement surgery of hip joint consists of the substitution of the joint with an implant able to recreate the articulation functionality. This article aims to review the current state of the art of the biomaterials used for hip implants. Hip implants can be realized with different combination of materials, such as metals, ceramics and polymers. In this review, we analyze, from international literature, the specific characteristics required for biomaterials used in hip joint arthroplasty, i.e., being biocompatible, resisting heavy stress, opposing low frictional forces to sliding and having a low wear rate. A commentary on the evolution and actual existing hip prostheses is proposed. We analyzed the scientific literature, collecting information on the material behavior and the human-body response to it. Particular attention has been given to the tribological behavior of the biomaterials, as friction and wear have been key aspects to improve as hip implants evolve. After more than 50 years of evolution, in term of designs and materials, the actual wear rate of the most common implants is low, allowing us to sensibly reduce the risk related to the widespread debris distribution in the human body.

## 1. Introduction

The hip is one of the most important joints that support our body, having the task of joining the femurs with the pelvis. The smooth and spherical head of the femur fits perfectly into the natural seat of the acetabulum, which is a cup-shaped cavity; the whole joint is wrapped in very resistant ligaments that make the joint stable. The hip joint is subjected to high daily stresses, having to bear the weight of the upper part of the body. Thus, especially with advancing age, these stresses can jeopardize its functioning.

Osteoarthritis of the hip is one of the most widespread alterations of the hip: it is a condition that causes intense pain due to a stiffening of the joint itself. The surface of the femoral head, due to arthritis, can undergo some alterations, becoming porous and causing damage to the entire joint complex. Osteoarthritis of the hip, as a degenerative pathology, involves irreversible damage due to which in many cases it is necessary to resort to the substitution of the compromised joint with an artificial one. A hip prosthesis is an artificial joint designed to perform the same functions as the natural one and which is surgically implanted. The surgical operation is referred to as Total Hip Arthroplasty (THA).

This paper aims to exhaustively review the state of the art of the biomaterials used as hip joint medical devices. More in depth, our review focuses on advantages, disadvantages and future perspectives regarding the use of biomaterials: polymers, metals, ceramics, and composites. This perspective may provide a clearer insight into how biomaterials research sets up the basis for the design of innovative devices for improved solutions to orthopaedic clinical problems.

### 1.1. History

Since its first application, the development of design and materials of hip prosthesis continuously progressed. Its development is one of the most challenging issues of the century in the field of implant technology [1]. Several materials were used for this scope: glass, polymers, metal alloys, ceramics, composites, etc., trying to combine biocompatibility and fatigue resistance, stiffness, toughness, withstanding static and dynamic loads, and high resistance to mechanical and chemical wear [2,3]. All these biomaterials were developed with the aim to improve the patient’s quality life, avoiding repeated surgery. First attempts at hip surgery date back to 1750, in England, willing to heal arthritis cases [4]. In 1840, the first idea of healing the hip was to replace it with a prosthesis [5]. This procedure was limited to resurfacing or replacing the acetabular part of the femoral head. To do so a wooden block was installed between the damaged terminal parts of the hip articulation. Due to wear particles released into the body, this procedure ended up being disastrous. Biological elements were therefore applied to solve the compatibility issue: skin, muscle tissue, pig bladder and gold foil [6]. Only several decades later were used different artificial materials, such as rubber, zinc, glass, wax and silver plates [4]. In 1880, Prof. Themistocles Glück implanted, for the first time, an ivory ball and socket prosthesis fixed to the bone by screws [7]. Later on, finding that human body could not accept large quantities of external material, he experimented with a mixture of plaster of Paris in combination with powder pumice and resin.

**Figure 1:**
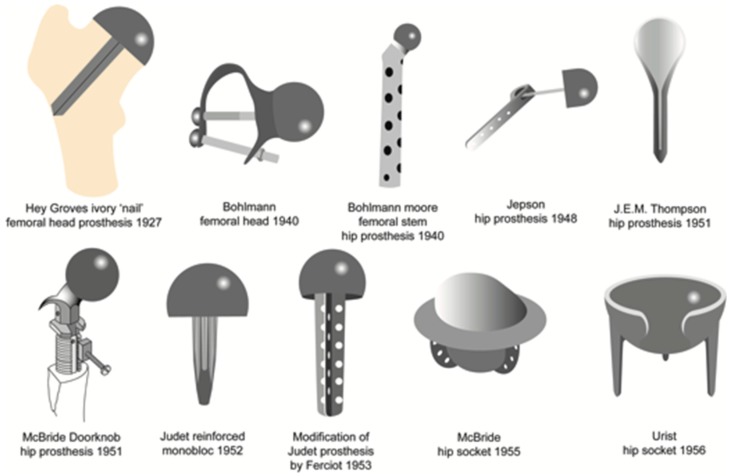
Evolution of the prostheses design.

Different materials were also introduced: in 1919, Delbet used rubber to replace a femoral head, whereas Hey-Groves used ivory nail in 1922 to simulate the articular surface of the femoral head [5]. In 1925, Marius Smith-Petersen introduced the first glass and bakelite femoral cup, defining the mold arthroplasty technique, that consisted of a hollow hemisphere adapted over the femoral head [8]. In 1938, Philip Wiles performed the first THA, employing a custom-made implant in stainless steel that was fixed to the bone tissue with screws and bolts. In 1950, Austin Moore introduced hemiarthroplasty, a new kind of hip implant, consisting of the replacement of the femoral head and part of the femoral neck using a long-stemmed element. The stem fitted into the femur cavity without cement, substituting around 31 cm of the proximal part of the bone, whereas the ball was placed on the hip acetabulum. This procedure was satisfactory, even though loosening of the implant was still a problem [5]. In Figure 1 are some of the mentioned hip prostheses designs.

**Figure 2:**
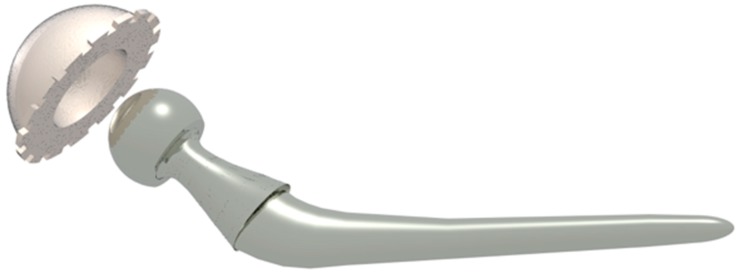
Charnley’s first LFA.

In 1960, the orthopedic surgeon, San Baw, started performing hip replacements, and in twenty years of work, over 300 ivory hip replacements, with an 88% rate of success [9]. The recognized pioneer of THA, as currently known, is believed to be Sir John Charnley. During the 60’s, he defined the concept of Low Friction Arthroplasty (LFA). His first prosthesis was made of a stainless-steel stem, fixed with acrylic cement, and a 22.2-mm diameter head coupled with a polytetrafluoroethylene (PTFE) cup, as shown in Figure 2. PTFE was unsuitable for prosthetic bearing, as it caused wear and tear that leaded to inflammatory reactions. To solve these issues, Sir Charnley adopted other polymer materials, such as high-density polyethylene (HDPE), and ultra-high molecular weight polyethylene (UHMWPE). He also used cement fixation for the acetabular cup [10]. With this combination, the wear effects were reduced, due to the smaller contacting surface and the hard-on-soft coupling. Sir Charnely made many variations to the original design of his LFA, which led to thousands of successful operations.

### 1.2. Current Materials

Four main types of bearings are studied and applied in THA: metal-on-polyethylene (MoP), metal-on-metal (MoM), ceramic-on-ceramic (CoC), and ceramic-on-polyethylene (CoP). Recently, hybrid combinations were introduced such as ceramic heads and metallic inserts (CoM) [11,12]. Many factors influence the choice between these types of bearing, such as the implant cost, age and activity level of the patient, complications during surgery, etc. MoM articulations were introduced first in 1950, by McKee and Farrar, leading to unsatisfactory results as two out of three implants were removed after 1 year due to loosening and the third removed for fracture [13,14,15]. After many improvements of the bearings, they were reintroduced in 1960, when the wear rate ranged from 1 to 5 mm^3^ per year (which was roughly 20 times lower than that registered for metal on polyethylene) [16,17]. MoM articulations were used for both total hip replacements and hip resurfacing (HR), which have the advantage of preserving the femoral head and neck, resulting in a less invasive operation and a lower dislocation rate. When, during the 2000s, the issues of metal debris came to light, the MoM replacements were almost stopped completely. In the early middle 2000s, these implants were used in more than one out of five cases in the UK and up to one-third in the US. Today, they are used in less than 1% of the total surgical operations [18]. MoM articulations have been used again in the last two decades, thanks to the appearance of new surface finishing techniques [6] that improve their wear resistance. On the other hand, MoM bearings aim to ensure high wear resistance, good manufacturability and low friction torque. However, even if lower wear volume is associated with such implants, very small particles are produced [19]. The amount of metal ions present in the serum and their potential toxic effects both locally and systemically are a cause for concern [19]. Moreover, polishing wear, promoted by wear debris, produced by the abrasive action of carbides, has been shown in retrieved Co-Cr alloy hip implants [19].

Up to the middle of the 1990s, the most widespread hip implant was MoP couples that worked well in older and less active patients [20]. Two relevant problems were still a concern: aseptic loosening as result of inadequate initial fixation caused by particle-induced osteolysis around the implant and hip dislocation.

In the 80’s, when aseptic loosening and osteolysis arose as main issues in metal-on-polymers hip implants, the firsts CoC couples were launched, starting with alumina and zirconia [21,22,23]. Zirconia ceramics have been introduced for orthopedic implants as a secondary ceramic material along with alumina for several years. Major advantages of ceramics for THA are their hardness, scratch resistance, and the inert nature of debris [24]. These characteristics promote the use of CoC bearings, and the inert nature of the wear debris result in them being the best choice for young patients. On the other hand, their use is expensive, and implants require an excellent surgical insertion to preclude chipping of contact surfaces.

The introduction of an innovative hybrid hard-on-hard bearing ceramic head and metallic insert claimed to reduce ion release and wear particle production and possibly the breakage of the ceramic insert rim [25,26,27]. In in vitro studies on CoM hip implants [12,28], smaller particles and lower wear have been found.

Nowadays hip joint prostheses are made with metals, ceramics and plastic materials. Most used are titanium alloys, stainless steel, special high-strength alloys, alumina, zirconia, zirconia toughened alumina (ZTA), and UHMWPE. Usually, stems and necks are composed of metals, whereas femoral heads can be both metal and ceramic, and the acetabulum can be made of metals, ceramics or polymers. There are several combinations that can be realized by using these materials with the aim of coupling with the fewest concerns and the highest long-term success odds.

Hereafter, we present an overall evaluation of biomaterials (polymers, metals, ceramics) for THA.

## 2. Polymers

Polymer materials were the first choice for low friction hip replacements, as proven by Charnely. Highly stable polymeric systems such as PTFE, UHMWPE or polyetheretherketone (PEEK) have been investigated due to their excellent mechanical properties and their high wear resistance. Nevertheless, when implanted, acetabular cups made of polyethylene generate debris that is attacked by the body’s immune system [29]. This leads to bone loss, also known as osteolysis; furthermore, since the debris accumulates in the area close to the implant, the bone loss leads to loosening of the implant stem. This results in the needs of a revision, namely, another surgery. Revision for loosening is four times higher than the next leading reason (dislocation at 13.6%) and is more severe in young patients [30].

### 2.1. PTFE

PTFE has a high thermal stability; it is hydrophobic, stable in most types of chemical environments, and generally considered to be inert in the body [31]. It was used by Charnley in his firsts THA, but exhibited two main drawbacks, which were found only after implantation in 300 patients [32]. The material had a very high wear rate, equal to 0.5 mm per month [33], and PTFE produced voluminous masses of amorphous material due to the vast number of foreign-body giant cells [34]. Furthermore, this debris elicited an intense foreign-body reaction that Charnley verified by injecting two specimens of finely divided PTFE into his own thigh [35].

Charnley tried to use a composite material based on PTFE reinforced with glass fibers (known as Fluorosint), finding poor performance in vivo, despite its fine behavior in vitro. The composite, after one year of implantation, developed a pasty surface that could be easily worn away. Plus, the filler acted abrasively and lapped the metal counter-face. Moreover, this composite material showed a higher rate of infection (20%) and loosening (57%) than the other materials employed [36].

### 2.2. UHMWPE

Charnley introduced UHMWPE in 1962, urged by the failure of PTFE as a bearing material and sustained by the promising behavior in laboratory tests [37]. The polymer is characterized by its excellent wear resistance, low friction and high impact strength. It is created by the polymerization of ethylene, and it is one of the simplest polymers. Its chemical formula is (–C_2_H_4_–)_n_, where n is the degree of polymerization, being the number of repeating units along the chain. The average degree of *n* is a minimum of 36,000 [38], having a molecular weight of at least 1 million g/mole as defined by the standard [39].

During the 1980s and early 1990s, aseptic loosening and osteolysis emerged as major problems in the orthopedic field, and these problems were perceived to limit the lifespan of joint replacements [40]. To limit the wear particle concentration and improve the overall mechanical characteristics, efforts have been made to improve the overall characteristics of UHMWPE for hip implants. In the 90s, scientists were able to correlate changes in the physical properties of the UHMWPE with the in vivo degradation of mechanical behaviors. UHMWPE was typically sterilized by gamma irradiation, with a mean dose of 25 to 40 kGy. This process resulted in the formation of free radicals, which are the precursors of oxidation-induced embrittlement. Only in the past decade did the radiation crosslinking achieve common diffusion. This process of crosslinking combined with thermal treatment has emerged to increase wear and oxidation resistance of the polymer, and a large number of laboratory and clinical studies indicated positive outcomes [41,42,43,44]. Crosslinked polyethylene is commonly abbreviated as PEX or XLPE. Currently, there are different treatments, including irradiation and melting, irradiation and annealing, sequential irradiation with annealing, irradiation followed by mechanical deformation, and irradiation and stabilization with vitamin E [45]. Crosslinking also affects the mechanical properties of UHMWPE, corresponding usually to a decrease in the toughness, ultimate mechanical properties, stiffness, and hardness of the polymer [46]. These factors could negatively influence the device performance in vivo [47]. Free radicals may form during the manufacturing process, allowing for oxidative changes in the XLPE. As a consequence, the wear resistance of the polymer is expected to decline, the opposite behaviour constitutes a sort of paradox. Muratoglu et al. [46] studied the wear behavior of UHMWPE, finding drastic changes as a consequence of crosslinking; these authors found that this process reduces the ability of molecules to orient and reorient, inhibiting this mechanism responsible for wear. It also appeared that the level of crosslinking, found in the study, overwhelmed the effects of reduced mechanical and physical properties in controlling the wear behaviour of UHMWPE. For the best outcome, XLPE should be cross-linked at a correct level of radiation, and then re-melted to remove the free radicals [48]. The exceeding free radicals that did not react to form cross-links through irradiation must be eliminated to prevent the formation of oxidized species and their recombination. The removal can be realized through two different methods: annealing or remelting; highly cross-linked polyethylene (HXLPE) has demonstrated superior wear resistance compared to gamma-sterilized materials [46]. By annealing below the peak melting point of the polymer, some of the crystalline regions are melted and the free radical concentration is reduced, but it is still measurable. On the other hand, through post-irradiation remelting, residual free radicals are reduced to undetectable levels, as measured by state-of-the-art electron spin resonance instrument. By this process, crystallinity is reduced after the melting step due to the hindrance by the new crosslinks, so the mechanical strength and fatigue resistance of the polymer decrease [49]. Several clinical studies have been realized on the in vivo oxidation of remelted or annealed XLPEs, even if our knowledge is restricted to what might happen during the first decade of implantation [50].

Muratoglu et al. [51] analyzed retrieved XLPE acetabular liners, finding minimal oxidation, but they discovered that the oxidation increases during shelf storage in air, producing severe damage. They assumed that two mechanisms could alter the oxidative stability of UHMWPE, the in vivo cyclic loading and the absorption of lipids. Lipids are able to react with oxygen and thus extract hydrogen atoms from the polyethylene chains, provoking the initiation of free radicals.

Rinitz et al. [52] investigated short- and middle-term retrievals made of remelted and annealed HXLPEs to determine whether oxidation can lead to mechanical property changes through oxidative chain scissions.

Their studies proved crosslink density decreases, corresponding to augmented oxidation for some highly cross-linked, thermally stabilized materials. Other clinical studies highlighted fast in vivo oxidation rates of post-irradiation thermally treated retrievals [53].

Successful outcomes are reached by HXLPE liners associated with a delta ceramic femoral head, as found by Kim et al. [54], finding an annual penetration rate of the femoral head of around 0.022 mm/year. Hamai et al. compared the clinical wear rates of annealed and remelted HXLPE liners by means of radiographs on 36 matched pairs of hip explants. They found significantly greater creep in the remelted than the annealed, but no significant differences between the steady state wear rates. The retrospective study of Takada et al. [55] compared the wear behavior between the second-generation annealed and first-generation remelted HXLPEs. Involving 123 primary THA, their study confirmed excellent wear resistance of both types of HXLPE, but found that second-generation annealed HXLPE had a better wear resistance than first-generation remelted HXLPE in a short-term follow-up. Also, D’antonio et al. [56] reported the wear rate of second-generation annealed HXLPE, which compared to a conventional polyethylene, represented a reduction of 72–86% (depending on other studies results). They further found a reduction of 58%, when comparing the linear wear of the second- and first-generation annealing HXLPE.

Crystallinity of the polymer is a function of the irradiation dose and of the thermal treatment [57]. Irradiation leads to smaller chains with augmented mobility, whereas the change in crystallinity after the thermal procedure depends on the temperature reached. If the treatment is realized below the melting point of 137 °C, the chain mobility rises, yielding higher crystallinity [58,59]. If the procedure is performed at higher temperature, the crystallization of the polymer, during the cool-down to ambient temperature, occurs in the presence of cross-linking, which decreases the crystallinity of the polymer and improve the wear resistance with small changes in toughness [58].

Basically, the mechanisms by which UHMWPE improves its chains occurs via plastic deformation of the polymer, with molecular alignment in the direction of motion that results in the formation of fine, drawn-out fibrils oriented parallel to each other [60]. As a result of this arrangement, the UHMWPE wear surface may strengthen along the direction of sliding, while it weakens in the transverse direction. In light of this, there is a will to realize reinforced polymers with high strength such as self-reinforced UHMWPE [61]. This composite is basically a non-oriented matrix of UHMWPE where reinforcement particles of the same material have been dispersed, resulting in a polymer with excellent biocompatibility, increased mechanical properties and the chance to be sterilized and cross-linked such as the traditional UHMWPE [61].

**Figure 3:**
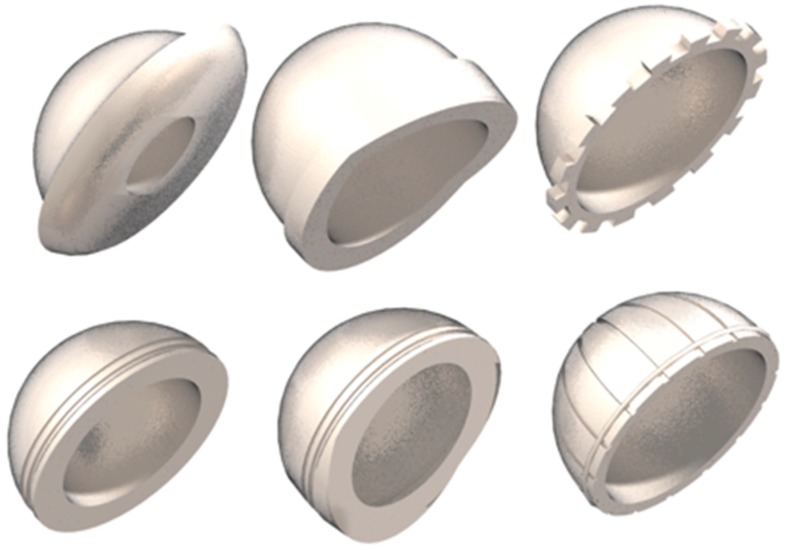
Some of the designs that are achieved with polyethylene for the acetabular cup.

In Figure 3 are presented typical PE prostheses designs.

In the recent years, a different approach was developed to stabilize polyethylene. Blending vitamin E with polymers was firstly meant as a hygienically safe stabilization, Tocopherol compounds were proposed as a stabilizer for polyolefin in the 1980s [62]. In 1994, Brach del Prever et al. [63] introduced UHMWPE blended with vitamin E for a prosthetic implant. In 2007, the first vitamin E-diffused, irradiated UHMWPE hip implant was clinically introduced in the United States (Biomet Inc., Warsaw, IN, USA) [64]. The blending led to the interruption of the oxidation cycle by decreasing the reactivity of the radical species, giving origin to a third generation of polyethylenes [64,65,66]. If vitamin E-stabilized, irradiated UHMWPE undergoes accelerated aging at high temperatures and/or in the presence of pure oxygen, it will be oxidatively more stable than gamma-sterilized or high-dose irradiated UHMWPE [67,68]. In vitro studies supported the hypothesis that vitamin E-blending would enhance the oxidative stability of XLPEs. There are also some drawbacks in the procedure: increasing the concentration of vitamin E in the blend is not viable, the obstacle of cross-linking in the presence of vitamin E prescribes the use of a lower concentration [69]. Therefore, a balance is needed to obtain elevate cross-linking density and high oxidative stability.

### 2.3. PEEK

Polyether-ether-ketone (PEEK) is a well-known biocompatible polymer used in orthopedic applications [70]. It has been considered as an alternative joint arthroplasty bearing material due to its favorable mechanical properties and the biocompatibility of its wear debris [71]. PEEK had been used as biomaterials, in particular in the spine, since the 1980s [72,73], due to its structure that confers outstanding chemical resistance, inertness, and thermal stability for in vivo conditions. In 1998, Wang and coworkers [74] tested acetabular cups made of PEEK on a hip simulator for 10 million cycles. They observed a reduction in the wear rate of almost two orders of magnitude in comparison to a conventional UHMWPE/metal or UHMWPE/ceramic couple. However, despite the good promises deriving from in vitro, low contact stress situations, when in high contact stress environments, there are questions about the suitability of this material as acetabular cups or knee tibial components [75,76]. No clinical data of its use are available.

## 3. Metals

Metallic materials have wide applications in the medical and bioengineering fields and are widespread as orthopedic implants components. The most common traditional metals used for THA are stainless steels, titanium alloys (Ti6Al4V) and—mainly—cobalt-chromium-molybdenum alloys. The latter have good corrosion resistance compared to other metals, and high toughness, high wear resistance and higher hardness (HV = 350) than other metals and polymers.

### 3.1. Cobalt Chromium Molybdenum Alloys

MoM articulation is typically produced from cobalt-chromium-molybdenum (CoCrMo) alloys. CoCrMo alloys are composed of 58.9–69.5% Co, 27.0–30% Cr, 5.0–7.0% Mo, and small amount of other elements (Mn, Si, Ni, Fe and C). These metallic alloys can be divided in 2 categories: high-carbon alloys (carbon content >0.20%) and low-carbon alloys (carbon content <0.08%) [77,78]. In addition, metallic alloys can be manufactured using 2 different techniques such as casting and forging; the grain size of the forged alloy is typically less than 10 μm, whereas the grain size of the cast material ranges from 30 to 1000 μm [79]. Intensive studies were done on the metallurgy for CoCrMo alloys with carbon; nevertheless, there is no complete phase diagram. This is mainly due to the complex phases existing in the system. Various carbide species, such as M_23_C_6_, and M_6_C can take place based on the heat treatment [80]. The differences in the microstructure of the carbides, their chemical composition, and nano-hardness are related to wear performances.

Cobalt and chromium are both present in the environment and in food. They are necessary to human beings as trace elements in the body but are toxic when highly concentrated. Patients with Co-Cr metal-on-metal pairings are exposed to wear with release of cobalt and chromium into the synovial fluid. These are capable of migrating to the blood before being expelled through the urine [81,82]. There is poor knowledge on the effects of circulating Co and Cr; they may affect mainly biological and cellular functions with potential effects on the immune system, mutagenesis, and carcinogenesis. In patients with metal-on-metal hip implant, elevated levels of circulating Co and Cr ions may be generated, and there is a positive linear correlation with a lymphocytic reactivity [83,84].

### 3.2. Other Metal Alloys

**Figure 4:**
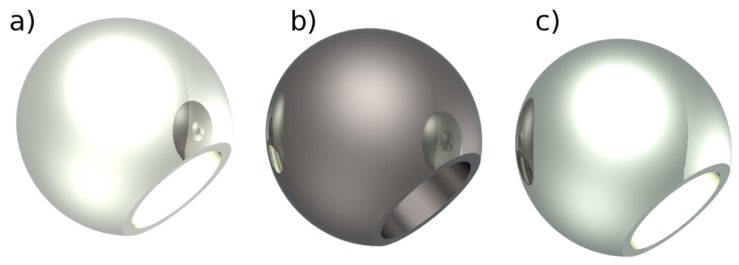
Metal femoral heads: (**a**) stainless-steel; (**b**) Oxinium; (**c**) CoCrMo.

Metallic materials have high module of elasticity, which limits stress distribution from implant to bone. Therefore, new metallic components have been developed with lower elastic modulus and higher corrosion and wear resistance. There is continuous research for new metallic alloys for application in hip prostheses to obtain a better biocompatibility along with superior mechanical properties. Still, it is mandatory to find a compromise between the many optimal characteristics desired for an implant material. Co-Cr-Mo alloys have low chemical inertness but high wear resistance, whereas stainless steel alloys have low strength and ductility. Zirconium (Zr) and tantalum (Ta) are refractory metals—due to their great chemical stability and elevate melting point—and are very resistant to corrosion, due to the stability of the oxide layer. As vanadium is a relatively toxic metal, some attempts were made to replace it in the widespread Ti-6Al-4V alloys. To improve biocompatibility and mechanical resistance, this Ti-6Al-4V alloys was replaced with iron (Fe) or niobium (Nb), realizing the improved alloys Ti-5Al-2.5Fe and Ti-6Al-7Nb. These alloys with respect to the traditional Ti-6Al-4V have greater dynamic hardness and lower elastic module, allowing a better implant/bone stress distribution. A new class of titanium alloys introduced into the orthopedic field uses molybdenum in concentration greater than 10%. Its presence stabilizes the β-phase at room temperature; these are referred to as β-Ti alloys. Having 20% less elastic modulus, they behave closer to real bones and have better shaping possibilities. Femoral stems made of a β titanium alloy have been used as part of modular hip replacements since the early 2000’s but were recalled in 2011 by the US Food & Drug Administration (FDA) due to elevated levels of wear debris. Yang and Hutchinson [85] found that the dry wear behaviour of a β titanium alloy (TMZF (Ti-12Mo-6Zr-2Fe (wt.%)) is very similar to that of Ti64, whereas their behaviour is completely different in simulated body fluid, where the wear of TMZF is significantly accelerated. Another recently introduced metal material is the oxidized zirconium (Oxinium, by Smith & Nephew), with a metal core and abrasion-resistant ceramic surface. The niobium alloy of zirconium has proven to decrease the UHMWPE wear rate and particle production considerably [86]. In Figure 4 it is possible to see the design of metal implants with different material renderings.

The revision rate of large head metal-on-metal and resurfacing hips is significantly higher than that of conventional total hip replacements. The revision of these bearings has been linked to high wear as a consequence of edge loading, which happens when the head-cup contact patch extends over the cup rim [87]. Underwood et al. [88] highlighted that using hip implants with low clearance, having more conformal contact and so a larger contact patch, increases the risk of edge loading and therefore intense wear.

## 4. Ceramics

The word ceramics derives from Greek, *keramos*, meaning potter or pottery. Ceramics were defined by Kingery [89] as “the art and science of making and using solid articles, which have, as their essential component, and are composed in large part of, inorganic nonmetallic materials”. It is likely to say that a ceramic is whatever material is neither a metal, a semiconductor or a polymer. Ceramics are used to build engineering components when wear resistance, hardness, strength and heat resistance are required. Ceramics were also defined as “the materials of the future”, as they are derived from sand that is about 25% of the earth’s crust as compared to 1% for all metals [90]. In the lasts decades, ceramic materials have exhibited great appealing and diffusion thanks to their chemical and physical characteristics, attracting the interest of biomedical scientists and companies [91]. Ceramic materials were introduced in the THA more than twenty years ago to overcome the major issue of polyethylene wear [92].

### 4.1. Alumina

Alumina was introduced in THA implants in 1971, when Boutin realized alumina-on-alumina hip coupling, leading to good clinical results [93,94]. Alumina ceramic has been one of the main ceramics to be used in THA, relying on its good tribological properties, meaning a favorable frictional behavior and a high wear resistance [95]. On the other hand, it has weaker mechanical resistance than other materials. It showed good performances in compression, but weak resistance to tensile stresses [96]. Alumina ceramics have been used in clinical applications for their tribological properties due to their hardness [97]. Among the ceramics, alumina is probably the most commonly used material.

The alumina used for hip replacements was different from the first generation of the material used for industrial applications. In particular, the first generation of alumina showed poor microstructure with low density, scarce purity, and large grain size. This generation of alumina was unsuited for biomedical use. The continuous efforts performed in this field allowed researchers to purify and improve this process, leading to an alumina for medical use, commercially known as Biolox^®^ [21,92]. The ISO 6474 standard, introduced in 1980, aimed to improve the quality of alumina for THA and to decrease the fracture occurrence. Alumina performance is related to different aspects, such as the density, the purity and the grain size. The last one, in particular, influences the wear rate, as it decreases with smaller grain size [92]. In the 90’s alumina hip implants were improved with the arrival of Biolox^®^ forte on the market, which could rely on innovations in the production process to furnish much better mechanical characteristics [21,92]. It was realized using improved raw material, with smaller gain size, low level of impurities and sintered in air. Biolox^®^ forte has a density of 3.98 g/cm^3^ and grain size of 3.2 m, whereas for Biolox^®,^ these values are 3.96 g/cm^3^ and 4.2 m [98].

Recently, concerns have been raised because of some clinical reports on the presence of audible noise in some ceramic-on-ceramic THA patients [99]. The so-called “clicks” or “grinds” have been described after THA, regardless of whether metal-on-polyethylene, metal-on-metal, or ceramic-on-ceramic bearings were used [100]. The “squeak” appears to be limited, however, to hard-bearing couples. It is probably related to implant design or cup orientation and the exact etiology of squeaking is the object of debates; there is neither a specific definition for post-surgery squeaking nor a universal categorization for the sound [101].

### 4.2. Zirconia

**Figure 5:**
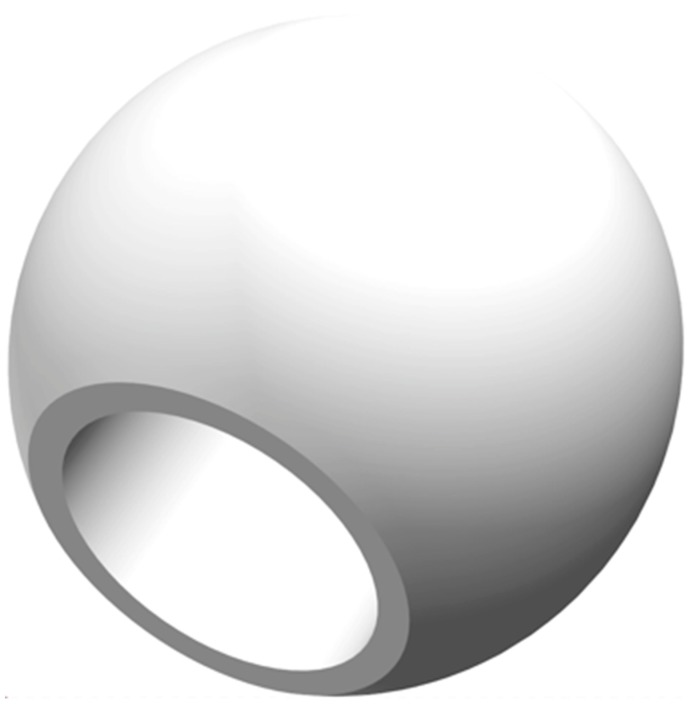
Zirconia femoral head.

Zirconia has high toughness and good mechanical properties; among all the monolithic ceramics, it has outstanding crack resistance [102]; these are the main reasons that made zirconia a very widespread alternative to alumina for THA. Firsts attempts were focused on magnesia partially stabilized zirconia (MgPSZ), that did not satisfy the wear resistance requirements [103]. Therefore, further developments were focused on yttria stabilizing oxide (Y-TZP), a ceramic that is completely formed by submicron-sized grains, representing the current standard for clinical application [104]. A picture of such a ceramic femoral head is shown in Figure 5.

Y-TZP is composed of tetragonal grains sized less than 0.5 μm, the faction of which retained at room temperature depends on the size, the distribution and the concentration of the yttria stabilizing oxide [96]. Such microstructural parameters define the mechanical properties of the Y-TZP. The tetragonal grains can transform into monoclinic grains, producing 3–4% volume expansion [105], which is the reason behind the toughness of the ceramic and its ability to dissipate the fracture energy. When a pressure acts on grains, e.g. a crack advancing in the material, they shift to the monoclinic phase, dissipating the crack energy in two ways: the T-M transformation and the volume expansion [106]. There are also metastable tetragonal phase particles, of which formation depends on grain size, stabilizing oxide concentration and matrix constraint. Above 100°C, the metastable particles in a wet environment can spontaneously transform into monoclinic particles [107]. As the transformation progresses, a decrease in material density and in strength and toughness of the ceramic can be observed. The structure of Y-TZP at room temperature is realized by submicron sized grains that grow during the sintering; it is therefore necessary to start from submicron size powders (e.g., 0.02 μm) and to introduce some sintering aid to limit the phenomenon [9].

With respect to metals, Y-TZP shows superior wettability properties that allows for fluid film formation between the articulating surfaces of an implant. Even if in clinical practice the Y-TZP femoral heads were only coupled with UHMWPE cups, tests performed on Y-TZP vs. alumina returned positive results [108]. From the wide investigation campaign on the wear performance of UHMWPE vs. zirconia, there is a general agreement on the fact that the wear is not higher than UHMWPE vs. alumina [109,110,111]. Discrepancies in results derive mostly from the differences in the bulk materials used in laboratories, in their finishes, testing procedures etc. There is great concern in the orthopedic community regarding the future of Zirconia as prosthesis. The market has decreased more than 90% between 2001 and 2002 (corresponding with the recall and abandon of Prozyr^®^, by Saint Gobain) [112]. More than 600000 femoral heads used in Y-TZP have been implanted worldwide, mostly in EU and US. The debate on the Y-TZP future is due to its pros and cons; it exhibits the best mechanical properties (resistance to crack propagation) but is prone to aging in the presence of water.

Zirconia manufacturers tried to shrink this problem, claiming that it was limited under in vivo conditions until 2001 when around 400 femoral heads failed in a short period. This event was related to accelerated ageing affecting two batches of Prozyr^®^ [112]. Even if the reason was identified to be processed controlled, this event led to catastrophic impact on the use of the Y-TZP, pushing some surgeons to go back to other solutions. The ageing problem and the Prozyr^®^ event are still an issue, and further efforts are required to gain confidence from the orthopedic society. In this way, the future seems to be based on the combination of zirconia and alumina to obtain advanced composites.

### 4.3. Zirconia Toughened Alumina

**Figure 6:**
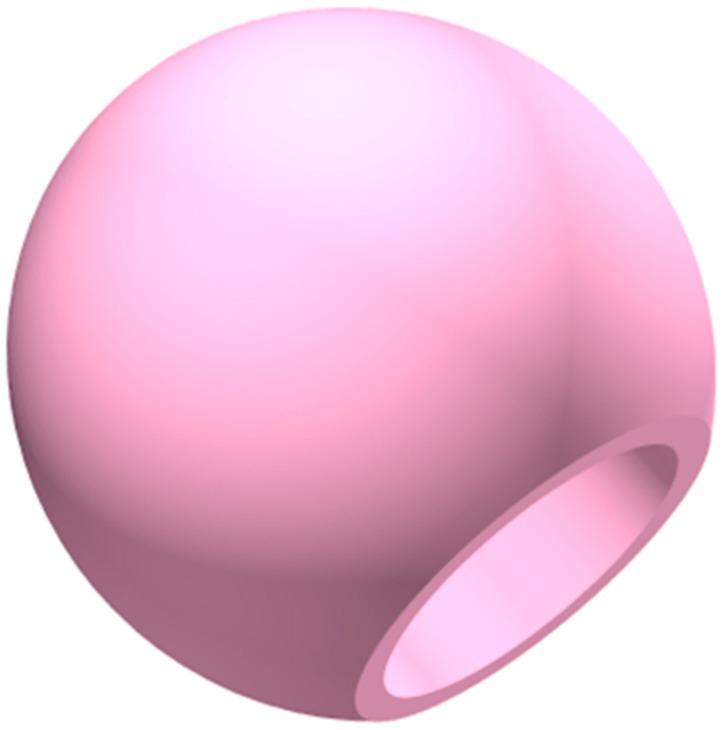
Biolox^®^ Delta femoral head.

In the second half of the 1970s, a new class of ceramic-based composite materials developed. This new composite material was realized by introducing up to 25% wt. of zirconia into an alumina matrix; this composite material is known as zirconia toughened alumina (ZTA). The addition of a fraction of zirconia to alumina results in a composite material of increased toughness [109,110,113]. In the 2000s, the first ZTA material introduced in a clinic was a composite known under the trade name of Biolox^®^ Delta [114]. A picture of such a ceramic femoral head is shown in Figure 6.

This material provides elevate resistance to the onset of cracking and to crack propagation [115,116]. This ZTA composite combines the best characteristics of both alumina and zirconia: the strength and toughness of alumina and the excellent wear resistance, chemical and hydrothermal stability of the alumina. This combination is realized through the uniform distribution of nano-sized particles of yttria-stabilized tetragonal zirconia (Y-TZP) in the alumina matrix. A small percentage of chromium oxide (Cr_2_O_3_) is added to counterbalance the hardness reduction caused by the zirconia presence. Strontium oxide (SrO) is added to the material, during the sintering process, to form strontium aluminate (SrAl_12–*x*_Cr*_x_*O_19_) platelets [117]. These flat and elongated crystals dissipate cracks energy and limit their advance, as it would require extra energy for the crack to overtake the crystal. The final composite is a mixture of roughly 75% alumina, 25% zirconia, and less than 1% chromium oxide and strontium oxide [96]. Deville et al. [118] found that Alumina Y-TZP composites exhibit significant ageing, but this process was far slower than usually observed in Y-TZP ceramics, which is ascribable to the presence of the alumina. On the other side, the presence of zirconia aggregates was recognized as the main cause of ageing sensitivity [119]. Realizing an optimal dispersion at acid pH can avoid the formation of zirconia aggregates, but as soon as the percolation threshold level (16 vol.%) is exceeded, ageing cannot be avoided.

These composites achieve a fracture toughness (K_IC_) up to 12 MPa·m^1/2^ and a bending strength up to 700 MPa. Due to the different elastic moduli of the two components, cracks will tend to move across the less stiff zirconia particles, inducing their T-M phase transformation that dissipates the crack energy.

## 5. Wear Behavior

Among the bearing surfaces involved in total hip arthroplasty, the biomaterials are submitted to sliding friction, producing particle debris, which, in turn, initiate an inflammatory reaction ultimately leading to osteolysis [120]. Wear is defined as a cumulative surface damage phenomenon in which material is removed from a body in the form of small particles, primarily by mechanical processes [121]. The wear mechanism is the transfer of energy with removal or displacement of material and in that follows an explanation of the mechanisms of wear observed with different biomaterials.

Pertinent literature was obtained from the Scopus database. The key words “hip joint replacement,” “hip prostheses,” “in vitro wear,” “in vivo wear,” and “THA” were searched in various combinations, and results were narrowed based on relevance to this review. Only articles from peer-reviewed journals were included.

### 5.1. Wear of Polyethylene

The primary mechanism of wear of polyethylene in THA is adhesive/abrasive, leading to the formation of sub-micron sized particles [33]. Elongated fibrils found in retrieved acetabular elements are precursors for this wear mechanism [58]. There is proof that the morphology of UHMWPE changes due to mechanical input. For example, it has been found that the mechanical properties of the polymer are dependent on both its crystalline and amorphous phases wear is led, at a micro-scale, by cyclic plastic deformation of the articulating surface [38]. Microstructural changes are correlated with plastic deformation in UHMWPE, in that lamellar alignment has been found during tests of cyclic tension, as well as decreased crystallinity in monotonic tension and compression specimens taken past yield [122].

There are different factors that influence the UHMWPE wear; some of them are related to the material itself, other are mostly due to the whole implant design. In the first category, is the nature or quality of the powder, as well as the tensile-rupture energy, the manufacturing process and the sterilization procedure. UHMWPE components can be obtained from ram-extruded bars; this process leads to internal inconsistencies or “dead zones”. The dead zones can lower the molecular weight and increase the wear rate of the final component [123]. Furthermore, the so-obtained elements tend to have micro-shred on their surface that can cause the third-body wear process. If the component is realized through heat stamping, as the melted outer layer cools, crystallization begins. The differential cooling leads to internal stresses resulting in a final element with anisotropic strength properties, vulnerable to oxidation degradation.

In the adhesion/abrasion wear mechanism, the surface conditions of the femoral head component, in particular its roughness and hardness, are key aspects. The hardness of the head material should be higher than that of the acrylic bone cement. If so, in a cemented arthroplasty, there will be less likely for third-body wear at bearing surfaces. To minimize the UHMWPE wear rate, the counter-body should be very hard and have a low contact angle (less than 70°); further, the head should be as smooth as possible and inert to oxidation.

### 5.2. Wear of Metals

The dynamic loading these implants undergo, together with the corrosiveness of physiological fluids can enhance the degradation processes. The combined effect of wear and corrosion does not consist of a simple sum of the two but more as a synergy realized between them called tribo-corrosion. Tribo-corrosion is defined as an “irreversible transformation of material in tribological contact caused by simultaneous physicochemical and mechanical surface interactions” [124]. In the last decades, a scaring occurrence of inflammatory reactions has been seen in patients with large head MoM THA, often with signs of tribo-corrosion at the head-neck interface. Tribo-corrosion arises not only at MoM bearing surfaces, but also at metal/metal modular junctions where micro-motions between the two components are possible.

More frequently, the wear of metal bearings can be distinguished in three main processes and their combinations: abrasive wear, due to either two or three bodies, adhesive wear and fatigue wear. However, other types of wear such as corrosive can occur. The corrosion resistance of metals relies on the passive layer formed on their surface in contact with a corrosive environment. Metals react with an oxygen-rich biological environment, realizing a thin protective oxidative coating – generally 2–5 nm thick – that limits corrosion. The oxidative layer forms immediately when exposed to in vivo conditions, but it does not last forever. Regarding the passive metals, wear can break the oxide layer on the surface, accelerating the dissolution of the base metal. The coatings can be scratched or rubbed off when surface contact happens. Even though the oxide layer spontaneously reforms, in restoring the protection of the surface, there is a rise in corrosion currents during the process, which causes the degradation of the material along with the release of metallic ions [125]. Once the film is worn out, the implant can release metal ions and particulates. The presence of these elements realizes third body wear that intensely increases wear rates. This damaging process applied on the coating, and metal ions released, and reformation of new coatings is known as *oxidative wear* [122]. The propensity of the layer to breakdown derives from the difference between the resting and breakdown potential. Regarding the CoCr alloys, the difference is high but corrosion can still happen under certain conditions. However, localized corrosion is not so common in CoCr alloys, which typically fail by trans-passive dissolution [125].

Galvanic corrosion can arise when different metals are in contact with each other, but also when the contact is between the same metal being partly under corrosion and partly under tribo-corrosion conditions. This type of galvanic contact is typical of modular implants, as in the neck-head contact.

Wear particles occurring in MoP implants are within the size range required for phagocytosis by macrophages, which is considered to be a cause of aseptic loosening [126]. On the other hand, particles generated by MoM implants belong to the nanometer scale, which reduces macrophage reaction. Nevertheless, the distribution of these particles within the body can have different biological effects and could be responsible for cytotoxicity, hypersensitivity and eventually carcinogenesis.

Investigations on retrieved 1st and 2nd generation MoM hip prostheses have shown linear penetrations of roughly 5 mm/year, which corresponds to a wear volume of approximately 1 mm^3^/year, two orders of magnitude lower than conventional polyethylene acetabular cups. The wear of hard-on-hard articulations such as MoM hip prostheses has two separate stages. Elevated bedding in the wear period occurs during the first million cycles or first year in vivo. Afterwards, a lower steady-state wear period occurs as the bearing surfaces have been subjected to the self-polishing action of the metal wear particles, which may act as a solid-phase lubricant. In vitro investigations, realized by hip simulators, generally show steady-state wear rates to be lower than those reported in vivo. The wear of tested MoM hip prostheses, 1 mm^3^/million cycles, is much lower than the more widespread polyethylene-on-metal bearings, 30-100 mm^3^/million cycles [19].

Each type of Co-Cr alloy has different characteristics that influence the wear rates of an implant. These properties comprise carbon percentage, manufacturing procedure and surface finishing. High carbon alloys have an initial wear of 0.21 mm^3^/million cycles for the cast implants and 0.24 mm^3^/million cycles for the wrought implants, whereas, alloys with a low carbon concentration have a significantly greater wear rate of 0.76 mm^3^/million cycles. The high percent carbon alloys show superior wear resistance as compared to the low percent carbon alloys with the assumption that there was no additional variation in other parameters.

In the human hip joint, wear can be designated as reciprocating sliding wear, because the contact area is smaller than the stroke of the wear path. Furthermore, the wear paths of the back and forth section of the cycle do not lie on the same geometrical lines, which lead to sliding wear. Even though, in sliding as well as in reciprocating sliding wear, all the other wear process—adhesion, abrasion, surface fatigue and tribochemical reactions—may be present at the same time [127].

### 5.3. Wear of Ceramics

Ceramic-on-ceramic implants have a life expectancy longer than implants with other combinations because of their very low wear rate. This clinical result led to the success of the ceramic implants: since 1990, alumina components were implanted more than 3.5 million times, whereas zirconia elements were used more than 600k times [128]. Nevertheless, ceramic is a brittle material and fractures can happen under adverse circumstances. Fracture probability is low (0.004–0.35% for alumina heads) but does occur [129]. The main causes of head fractures are local stress concentrations that are ascribed to taper interface contamination or damage or to loosening of the head on the taper [130,131].

Affatato et al. [113] tested different ceramic configurations, i.e., pure alumina vs. alumina composite. The wear rate was lower for the pure alumina than for the alumina composites. Still, no statistically significant differences were observed between the wear behaviours of these materials at a 95% level of confidence. In different work, Affatato and co-workers [11] carried out wear tests to compare the tribo-behaviour of different sizes of ceramic components. Two different batches of alumina Biolox^®^ Forte (28 mm vs. 36 mm) were tested on a hip simulator under bovine calf serum for five million cycles. They found that the 36 mm Biolox^®^ forte size showed less weight loss than the 28 mm Biolox^®^ Forte size.

Nevelos et al. [132] studied the behavior of CoC bearings realized with hot isostatically pressed alumina and compared with the standard alumina ones. They found a reduction of the wear rate for the hot-pressed prosthesis when working under standard conditions. Different behavior was observed under Gelofusione^®^ (4% w/v solution of succinylated gelatin) and water lubricants, where the non-hot-pressed ceramic showed a lower wear rate. Even so, the results were significantly affected by uncertainties as testified by the large error bars. It is worth noting that the wear rates reported by the authors, under standard testing conditions, were an order of magnitude lower than the majority of reported clinical wear rates for in vivo ceramic prostheses [133,134].

A summary of the in vitro tests realized on the different combinations of materials is presented in Table 1 and Table 2, for soft and hard bearings, respectively.

## 6. Discussion

Since its first application, THA has evolved in both terms of material and design. After a first experimental phase, that went along many failures, the UHMWPE was established as the most widespread material to be used as acetabular component. The arrival of CoCrMo destabilized its supremacy for a while but the combination of the two resulted in great pairing. Ceramics are the most recent materials introduced in the orthopaedic field, having the best tribological behavior, they rapidly achieved great success. During the 1970s and 1980s, the great majority of hip prostheses in clinical use incorporated a polyethylene acetabular liner bearing against a femoral ball of metal or ceramic. The willing to resolve the issues of hip implants pushed many researchers to study the various combinations of materials and to introduce some variation of their characteristics. These alternatives included highly cross-linked, thermally stabilized polyethylenes against metal, composite ceramics. The latter composites realized with ceramic matrix are the most successful ones.

The biomaterials used in the orthopedic field play a vital role, and their validation through in vitro tests is of paramount importance. The main objective in the field of biomaterials for hip implants is the reduction of failure incidences. We believe that knowledge of wear rate is an important aspect in the pre-clinical validation of prostheses. Wear tests are executed on materials and designs used in prosthetic hip implants to control their final quality and obtain auxiliary knowledge on the tribological processes. Researchers should not forget that other issues still impact the life expectancy of the prostheses, such as the sensitivity of the cup position and edge loading in ceramic bearings. Therefore, several steps forward are required to improve the overall performance of the implants, such as the ability to sustain high demand activities—for young patients—and preserve the bone from retro-acetabular loss.

New implant concepts, such as hip resurfacing and shorter cementless hip stems, are today mostly used in Europe and may also influence the future of hip arthroplasty. Considering that the number of patients who undergo total joint arthroplasty, and consequently revision, is increasing due to an aging population, patients remain the principal players in this process. There is also an increase in the economic health expense, so it is necessary to reduce the number of revisions to reduce these costs. Knowledge of the behavior of individual prostheses in certain clinical conditions may help in this matter. Nowadays there are many prosthetic models on the market and few scientific evidence of good methodological quality to support the use of most of them. Under these conditions, it is difficult to monitor the use of prosthetic devices and ensure the traceability of the patients in the case of adverse events.

Many countries are adopting a registry for post-marketing surveillance in order to collect data on joint prosthetic performance. Registries can be compiled at the international, national or regional level but also locally, such as in hospitals [148]. Through the registers, it is possible to evaluate the effectiveness of an implant, its lifetime and performance for the treatment of specific cases. Registries are an important tool for research; they allow the identification of patients with a certain condition or outcome for prospective observational studies of large size. In this way, the registry can educate the surgeon to select the best type of prosthesis and surgical technique. Consequently, the healthcare resource will be properly used.

## 7. Conclusions and Future Prospects

The future of total hip replacement should be perceived as a divergent tendency for developed and developing countries. Advances in technology, improved materials and better understanding of natural tissue reactions will certainly result in breakthroughs of implant selection. Due the ageing of the population, the number of joint replacement surgery has increased in the last years [149]. Consequently, also the number of revision surgeries is growing, as the life expectancy of patients is longer than that of prostheses [150,151].

Current trends in prosthesis design emphasise the use of biocompatible materials that are strong enough to withstand the more active lifestyles of many patients, whilst generating minimal wear debris. As the main issue affecting the long term durability of prosthesis is wear and the propagation of wear particles, vast research is currently being undertaken to improve such biomaterials to give an “infinitive prosthesis life”. Analysis of component wear is therefore essential for future progress; retrieval analysis of a well-functioning bearing prosthesis could help in improved the biomaterials. Controversy regarding the safety of metal-on-metal bearing surfaces still remains, particularly in relation to metal ion release and potential hypersensitivity reactions [152,153,154]. Ceramic-ceramic implants have been demonstrated to provide the lowest wear rates in comparison to other material options possible for ceramic-on-ceramic THA [9,98,155]. Trends in material development are also strongly influenced by the desire to improve hip function and stability through the use of increased head diameters [97]. Today, there is a large number of prosthetic models on the market and limited scientific evidence of good methodological quality to support their usage; the expected costs of treatment in a decade perspective amount to a fraction of what they turned out to be. Worldwide, countries should develop strategies to tackle the problem of increasing demand for medical services in a more simplified and inexpensive way, as they may not even be capable of absorbing the technology in the absence of infrastructure, lack of training and know-how. Prevention, i.e., appropriate dietary and lifestyle modifications, may be important to reduce hip implants. In addition, as mentioned above, countries should adopt registries for post-marketing surveillance. Such registries should collect all data on joint prostheses performance in order to evaluate the effectiveness of an implant, its lifetime and performance for the treatment of specific cases. In this way, the registry can educate the surgeon on the best type of prosthesis and surgical technique or to improve preoperative planning [3]. Consequently, the healthcare resource will be properly used. In conclusion, based on the increase in hip implants in young and older patients, the development of new biomaterials correlated with the lower wear-rate, and the systematic collection of limited essential information on the surgery and the definition of a single endpoint, the failure of the system and its replacement, allow us to monitor the device over time after its market introduction. This may help the surgeons to improve the quality life of the patient in the near future.

## Figures and Tables

**Table 1:** Soft bearings’ wear rates found *in vitro* through simulators.

Soft Bearings	Paired Materials *	Overall Wear Rate (mm^3^/Mc)	Ref.
MoP	CoCr—XLPE	6.71 ± 1.03	[135]
	Biolox®Delta—XLPE	2.0 ± 0.3 **	[136]
CoP	CoCrMo—XLPE	4.09 ± 0.64	[137]
	Alumina—XLPE	3.35 ± 0.29	[138]
	Alumina—PE	34	[139]
	ZTA—PE	80	[140]

* all the abbreviations are reported at the end of the manuscript. ** only in this case the unit of measure is mg/Mc.

**Table 2:** Hard bearings wear rates found in vitro through simulators.

Hard Bearings	Paired Materials *	Overall Wear Rate (mm^3^/Mc)	Ref.
CoM	CoMplete	0.129 ± 0.096	[141]
	Biolox^®^Delta - CoCrMo	0.02 ± 0.01	[142]
	Biolox^®^Delta-CoCrMo	0.87	[28]
CoC	Biolox^®^Forte-Biolox^®^Forte	0.052	[28]
	Alumina-Alumina	0.03	[143]
	ATZ-ATZ	0.024	[143]
	ATZ-ATZ	0.06 ± 0.004	[144]
	ZTA-ZTA	0.14 ± 0.10	[144]
	ATZ-ZTA	0.18	[145]
	ATZ-Alumina	0.20	[145]
	Alumina-Alumina	0.74 ± 1.73	[144]
	Biolox^®^Delta-Biolox^®^Delta	0.10	[146]
MoM	CoCrMo-CoCrMo	0.60 ± 0.18	[142]
	CoCrMo-CoCrMo	0.11 ± 0.055	[147]

* all the abbreviations are reported at the end of the manuscript.

## Abbreviations

Alumina toughened	ATZ
Ceramic-on-ceramic	CoC
Ceramic-on-metal from	CoMplete
Cross-linked	XLPE
Metal-on-metal	MoM
Metal-on-polyethylene	MoP
Polytetrafluoroethylene	PTFE
Polyetheretherketone	PEEK
Total hip arthroplasty	THA
Ultra-high molecular weight polyethylene	UHMWPE
Zirconia toughened alumina	ZTA
